# Medical students' perceptions in relation to ethnicity and gender: a qualitative study

**DOI:** 10.1186/1472-6920-6-17

**Published:** 2006-03-08

**Authors:** Heidi Lempp, Clive Seale

**Affiliations:** 1 Academic Department of Rheumatology, King's College London School of Medicine at Guy's, King's College and St. Thomas' Hospitals, Weston Education Centre, Cutcombe Road, London SE5 9PJ, UK; 2 School of Social Sciences and Law, Brunel University Uxbridge, Middlesex, UB8 3PH, UK

## Abstract

**Background:**

The British medical student population has undergone rapid diversification over the last decades. This study focuses on medical students' views about their experiences in relation to ethnicity and gender during their undergraduate training within the context of the hidden curriculum in one British medical school as part of a wider qualitative research project into undergraduate medical education.

**Method:**

We interviewed 36 undergraduate medical students in one British Medical School, across all five years of training using a semi-structured interview schedule. We selected them by random and quota sampling, stratified by sex and ethnicity and used the whole medical school population as a sampling frame. Data analyses involved the identification of common themes, reported by means of illustrative quotations and simple counts.

**Results:**

The students provided information about variations patterned by gender in their motivation and influences when deciding to study medicine. Issues in relation to ethnicity were: gaining independence from parents, perceived limitations to career prospects, incompatibility of some religious beliefs with some medical practices and acquired open-mindedness towards students and patients from different ethnic backgrounds. Despite claiming no experiences of gender difference during medical training, female and male students expressed gender stereotypes, e.g. that women bring particularly caring and sympathetic attitudes to medicine, or that surgery requires the physical strength and competitiveness stereotypically associated with men that are likely to support the continuation of gender differentiation in medical careers.

**Conclusion:**

The key themes identified in this paper in relation to ethnicity and to gender have important implications for medical educators and for those concerned with professional development. The results suggest a need to open up aspects of these relatively covert elements of student culture to scrutiny and debate and to take an explicitly wider view of the influence of what has sometimes been called the hidden curriculum upon the training of medical professionals and the practice of medicine.

## Background

In the last four decades the medical student population in the UK has diversified in terms of gender and ethnicity [1-3] but less so in relation to social class[4]. Since 1965 the proportion of women entering UK medical schools has risen from 20% to almost 55% [5]. It is predicted that the proportion will plateau at 60%–65%, similar to many other caring professions [5], even rising to 70% at some universities [6]. In a parallel development, in some medical schools nearly 40% of the student intake is from ethnic minorities [3]. White men now comprise just 26% of all UK medical students [1]. While Asians comprise 7% of the UK population, they accounted for 19% of medical students in 2001[1] although Black Caribbeans remain under-represented [1,7]. In London the proportion of Asian students is particularly high [8]. By 2002, medical school graduates were made up of 72% 'whites' and 28% 'non-whites' [1].

At the same time, attempts to further widen the participation of students from diverse social and ethnic backgrounds are being introduced[9]. Even so, some studies point to the existence of gendered and restricted circumscribed areas of practice, including a gendered and ethnic 'glass ceiling' (which the Oxford English Dictionary defines as 'an unofficial or unacknowledged barrier to personal advancement, especially of a woman or a member of an ethnic minority in employment') in the medical profession [4,10-15]. Other key themes have been identified in this domain of research. In relation to gender: harassment, [16-19] the lack of female role models, [15,19-21], gender stereotyping [14,15] and less access to patients during obstetrics and gynaecology attachments for male medical students and for female medical students during surgery clerkships [22,23] have been highlighted. In relation to ethnicity: poorer performances in some ethnic groups than in 'white' students in clinical examinations, despite good written examination results has been found [24] as well as differences in communication style during objective structured clinical examinations [25].

Against this background we have conducted a qualitative study in one medical school in the UK to examine students' experiences and perceptions in relation to gender and ethnicity. We have extended our earlier work on the hidden curriculum of the medical school [26]. This has been defined as 'the processes, pressures and constraints which fall outside ... the formal curriculum, and which are often unarticulated or unexplored' [27].

## Method

The study cohort consisted of 36 students in years 1–5 from one medical school in the UK (see Table 1) in 2000. We stopped the recruitment when saturation was reached for the key themes, meaning the point at which no new themes emerged, acknowledging that this is a subtle judgement. The students were selected by random and quota sampling, stratified by gender and ethnicity using the whole student population of the medical school as the sampling frame. 13 students refused to participate in the study.

Qualitative data were collected in one-to-one semi-structured interviews (by HL), and conducted in a private room in the medical school (see Appendix 1), and each interview took an average of 50 minutes. Following the transcription of the interviews, we conducted a qualitative thematic analysis [28] with simple counting[29], supported by the Nvivo and Concordance software programmes. Themes were identified by reading and discussing the data and formulating a coding scheme that was used to assign passages of text to the themes. In our report for this paper we describe each 'theme' (for example, 'interest in people' as a reason for studying medicine), state how many interviewees expressed it (e.g. 28/36) and, for more important themes, provide an illustrative quotation. Our counting procedures are designed to give a rough idea of the prevalence of the themes in the data. They are not intended to provide a basis for statistical generalisation to a population.

The interview schedule (see Appendix 1) contained a number of key questions in relation to women in medicine, all of which were asked of each student. In reference to ethnicity, no specific questions were included, but relevant information provided by students voluntarily from all parts of the interviews was used in the analysis. We sought to improve validity by close examination of the plausibility of the accounts in the experience of the authors, asking for further clarification and examples of key points during the interviews, and by searching and accounting for negative instances in the data analysis [30].

Each medical student provided written informed consent prior to their study participation. Formal permission for conducting the interviews was obtained from the education committee of the medical school where the study was undertaken. The full requirements for the ethical conduct of the research, as set out by the British Sociological Association, were strictly complied with.

## Results

### Altruism vs. pragmatism

Students' choice of medicine as a career was influenced by a number of factors, the most common of which were an interest in both people (28/36) and science (19/36). Female students were more likely to mention 'being with people' as an 'internal' (private) motivating aspect (19/21 compared with 9/15 males) and were dissimilar to some male students (7/15) who reported 'external' (public) reasons why they decided to study medicine.

'I really love communicating with people and I really get on well and I like talking to people and that is when I decided to do medicine rather than veterinary [medicine like my father], yeah that was really the main factor' (Year 2 'white' female student).

'It [medicine] has a lot of social importance and that it was a very big position to be in....you are making a change to somebody's life that perhaps no other ordinary person would be able to do...'(Year 5 'non-white' male student).

External career incentives were not mentioned by any of the women. Only female students (7/21) expressed a fascination with the human body (anatomy) and its functions.

'I have always been interested in the anatomy side of things, I mean to me there is nothing more amazing than how the human body works and how the sperm and an egg meet and make a unit. To me that is amazing....' (Year 2 'non-white' female student).

Half of the students (18/36) reported how older male 'informants' (such as medical relatives) had influenced their decision to study medicine, whereas few (4/36) mentioned such female 'informants'.

'I wasn't sure if I wanted to do medicine, and my mum's friend offered to give me some work experience in hospital. I went down, and I thought it was really good. Like *he *was a really good doctor and I was really impressed, so I thought I'd apply for it. And that was really what happened.' (Year 3 'white' male student).

'And I guess the other person is probably my mother, mostly. Just because of getting into medicine when she did, and doing as well as she did. Yeah, so... so like, you know, she was so...very, very dedicated.' (Year 1 'white' female student).

### Ethnic and gender stereotypes

A number of perceived differences and disadvantages were reported by 'non-white' students. The issue of independence from family was mentioned by 8 of the 14 'non-white' students. They reported conflicting loyalties between the culture of their families and the demands of both academic work and social life at the medical school. Such concerns were not stated by any 'white' students.

'I think the major thing is compromising my family life... which is in my religion it's.....you know, families are very important....clearly it is less in the Western culture but....much so in my culture...compared to everyone else in my family like my brothers they're able to go to things ... a lot of the time I can't go home at the weekends although my parents are expecting me to. When I am at [clinical] work coming home is much harder.' (Year 3 'non-white' female student).

Two female black (African) students identified a contrast between their image outside the medical school and their identity as future doctors.

'People sort of say, "Oh my daughter tried to get into medical school and she didn't," and it's kind of...you know, I do feel that I do have to sort of justify my intelligence all the time. Umm, I don't know. I suppose...I don't know, I suppose because, okay, I'm black, I'm female – and also, I suppose, I don't particularly look kind of studious or whatever. People don't think...people just don't think I fit the image. And I do find myself trying to conform, and be a different person sort of in hospital, so I look like a medical student.' (Year 4 'non-white' female student).

Such perceptions of needing to conform to the expected template of a 'real' medical student were not brought up by any 'white' or Asian students. In this context the lack of 'non-white' role models may be significant. It is telling that this issue of not fitting in was raised by a black female student, who in a sense was furthest away from the traditional stereotype of a white, male doctor [26]. Some 'non-white' students (5/14) were concerned about the potential for their ethnicity to adversely affect their career prospects in medicine.

'There is something about doctors and position of power,...it's just so obvious ...consultants are all Caucasian. And registrars are mostly all, umm, Asian – it's an amazing clear-cut line, you can actually see it!' (Year 4 'non-white' female student).

'People get to places [in medicine] because of who they know, and I don't appreciate that. Part of the reason I probably don't appreciate that is because I'll never be part of the old boys' network fully, and I think that's because of my ethnic origin.'(Year1 'non-white' male student).

Among female Muslim students, 5 out of 6 mentioned the importance in the Islamic religion of separating men and women in clinical practice, a matter which they said did not always receive enough attention during the training.

'I know this girl [medical student] who was placed with a male GP and she said to him that she couldn't talk to his male patients while she was there with the doors closed and...this would be ladened [sic] by her religious beliefs. Religion... has a big role to play in medicine, because I am a Muslim.' (Year 1 'non-white' female student).

Five of the whole sample of students said that shy and quiet female Asian students were more likely to be humiliated or ignored by consultants during the ward rounds.

'The consultants do go for more insecure...I mean some students are less able clinically than others, but they tend to pick on those who are obviously less able.... particularly some of the quieter Asian girls, I mean they don't look fragile, but are mentally fragile, you know, they are not very kind of aggressive, a lot of them I think, you know wouldn't take that [humiliation], couldn't take it, they wouldn't know how to deal with it.' (Year 4 'white' female student).

At the same time both 'non-white' (4) and 'white' students (6) acknowledged that through the study of medicine they had benefited from being in contact with a wider range of ethnically diverse students (and patients).

'Coming to the medical school was a real eye opener to me.....especially this year, I'm with a lot of Muslims and people from a different religion. And it's amazing where I come from, it's 22,000 [the population] but I'd never spoken to an Asian person or a black person before I came here, so that's a big change for me. And learning about what they think, and their...how they, er, learn about medicine; their views and what they're going through.' (Year2 'white' female student).

### Gender and future careers

The majority of students (22/36) commented on the fact that female medical students are now as common, or are more common, than males. Twenty one noted that there are more male than female clinical consultants and tutors.

'The top lecturers, I mean you can never see the dean of the medical school ever being a lady. And when you see this busts and paintings, they are always male.....We do have Prof. XX she is a lady, but generally it does tend to be still I think pretty male dominated on the top. I would say it is about....75% men.' (Year 2 'non-white' male student).

Nevertheless, in response to direct questioning (see Appendix 1) 29/36 denied any gender differences in the training experiences of themselves or their fellow students. Elsewhere in the interviews, though, some discrepancies were reported. For example, 10/21 female students and 1/15 males remarked that the people they regarded as positive role models treated them with respect.

'She treated students not like idiots, good manner and communication with patients which I could learn from.' (Year 3 female student).

In relation to medical specialties, 7/36 students (4 female, 3 male) commented that they had observed or experienced direct difficulties related to gender during the obstetric and gynaecology rotation, where male students had limited opportunities to obtain practical skills since female patients were reluctant to be examined by male students.

'I did feel at a disadvantage doing obstetrics in that a lot of women are not prepared in this day and age to allow you to attend their delivery or actually do their delivery. Er, that was pretty frustrating at the time; and in gynaecology as well.' (Year 5 'white' male student).

A majority 22/36 (14 females, 8 males) stated that surgery was dominated by men, reporting their perception that the specialty required physical strength (4 females/2 males), competitiveness (4 females/1 male), unusually hard work and long working hours (1 female/4 males) in order to succeed. Nevertheless four female students (of 21) were considering surgery as a career option, and four male students (of 15).

'Yes, top jobs, still tend to go to men, big surgeons, yeah. All anatomy demonstrators want to be surgeons, and 90% of them are male. It is all an assumption really. But I think it got some truth to it.' (Year 2 'non-white' male student).

'I think women who want to be surgeons, I think good luck to them... I think some jobs are more suited...like orthopaedics is not really a woman's job to do... it is quite difficult, because you need to be strong to move bones and the operations are quite...plumbing... carpentry...'(Year 3 'white' female student).

Most students (23/36) identified certain specialties as being 'suitable' for women, these being (in descending order of frequency) obstetrics and gynaecology, general practice, paediatrics and palliative care. Many (20/36) also described qualities which they believed women bring (positively) into medicine, such as talkativeness, empathy, caring, ability to listen, and emotional expressiveness.

'From a man's point of view, men can do cardiac thoracic, so we can be leading a cardiac thoracic surgery, possibly I think if the ladies thinking of having a family, personally I think they should choose something that, would fit their sort of setting, their life for example, obs and gynae.' (Year 4 'non-white' male student).

'Women are better at caring and communicating, they are thoughtful.' (Year 3 'white' female student).

Eleven students (7 females, 4 males) used the word 'sacrifice' in relation to women and their medical career, for example in having to limit either their career or their family aspirations, but none used this remark to describe the careers of male doctors.

'I think if you have a special interest in something...it depends how much of your personal life you are willing to sacrifice. If you want to do surgery then I don't think you can really have a very good family life, in the sense that the hours are very long. If you want to get to a consultant level you are going to have to sacrifice lots of things to get there.' (Year 4 'non-white' female student).

## Discussion

As this study is based on the observations and perceptions of medical students in a single medical school in the UK, the extent to which the findings are generalisable to other medical schools remain to be established. Elsewhere [29] we have reviewed various approaches to generalising from case studies such as this. The qualitative aspect of our accounts is designed to produce a degree of contextual detail that will help readers assess the degree to which our findings are transferable to other settings. We recognise though, that empirical generalisability can only firmly be established by doing similar research in other medical schools.

In this medical school, at the point of entry, there were clear gender differences in students' reasons for this career choice, with men more interested in 'external' factors related to the expectations of others for example, and women in 'internal' issues such as an interest in finding out more about the functioning of the body. The influences of mainly male 'informants' on career decisions reflects the reality of a profession still dominated by males in most senior positions.

'Non-white' students' difficulties in achieving independence from parents reflect cultural differences in family relationships. Some 'non-white' students identified a discrepancy between the ethnic composition of the medical student intake and the ethnic composition of the medical profession, suggesting that this could have negative implications for their own career prospects, as alluded to in previous publications. [10,11] The experiences of black (African) female students may be different again, and this requires further research with a targeted sample of this minority population in medical schools. Accounts by female Muslim students implied that religious beliefs were not always acknowledged by teachers. The ethnic diversity of medical students was welcomed by some students among all ethnic groups.

When asked in general terms about gender differences in their medical training, most female and male students claimed that these did not exist, which supports the finding of Gjerberg who showed similar proportions of male and female students intended to enter surgery [15]. Nevertheless the data analysis in relation to more specific aspects of their training did reveal several ways in which such gender effects were identified by the students. Self-contradiction of this sort is quite normal in qualitative interviews (as it is in everyday life) and is worth noting in itself as a finding. Interestingly, having denied that such gender differences exist, when male and female students did disclose examples of such gender-related distinctions in their accounts, they did so in terms that were to a large extent consistent with traditional gender stereotypes, as has been shown in previous reports [12-16].

Almost a third of students (more females than males) insisted that the future of women in medicine, but not for men, consists of a harsh choice between home responsibilities and career. This does not bode well for the future family life of these doctors. Finally, the perceived reluctance by female patients to be examined by or in the presence of male medical students during gynaecology outpatient clinics, identified by a fifth of participants, has been confirmed by other reports [31].

## Conclusion

Many of the issues raised by these students are somewhat sensitive and may be especially difficult to challenge. The key themes identified in this paper in relation to ethnicity and to gender have important implications for medical educators and for those concerned with professional development [16,32,33]. The results suggest a need to open up these relatively covert aspects of student culture to scrutiny and debate. This will mean taking an explicitly wider view of the influence of what has sometimes been called the hidden curriculum [26,27] upon the training of medical professionals, and upon the practice of medicine [34,35].

## Appendix 1: Semi-Structured Interview

### Introduction

• first of all, can you tell me...

• why did you decide to study medicine?

• any event leading to it?

• at what age did you decide to study medicine?

• has the medical training met your expectations so far?

• if yes, why?

• if no, why?

### Training

• what do you enjoy most in your training? (and why?)

• what do you enjoy least in your training? (why? or, what is difficult?)

• do you think there is a balance struck in the medical training between the technical/diagnostic skills and a caring approach towards patients?

• if no, can you provide examples/expand?

• how do you succeed in your training? (if not, why not?)

### Impact of training on individual medical student

• what compromises do you have to make because of your demanding training?

• who have you turned to in times of difficulties with your study?

• can you describe what happened?

• who have you turned to in times of (serious) personal difficulties since starting medical training?

• can you describe what happened?

• how did the teaching of anatomical dissection affect you at the very beginning of your training? Was it a powerful experience?

• how has your identity been affected since you started your training (e.g. conformity, change how? different how?)

• how has the medical training changed your view of the world?

• how does teaching differ from your experience when you went to school?

• can you describe the difference?

• have your relationship(s) with family and friends changed since you started medical training?

• if yes, how and why do you think that is ?

### Professional relationships

• who have you admired (role model) during your training?

• can you describe what you admired (who/why/when/where?)

• do students give staff a hard time during the teaching?

• if so, how/or what happens?

• what do you like/dislike about your contact with patients?

• (relationship with patients)

### Women in medicine

• in your experience are there still any gender differences within the medical profession?, i.e.

• are the training experiences of female medical students different from male medical students during the studies? (can you give examples, how, why?) (or, are students treated equally during the training?)

• do you think that women are more suited to certain kinds of medical specialities than men? (which/why?)

• do you feel respected as a medical student in the class room/clinical setting (by patients, other professionals, doctors?)

• if no, why not, can you describe what happens?

• if yes, can you describe what happened? (or have you been humiliated/best or worst learning experience?)

• in your opinion, what are the likely scenarios for the future of women in medicine?

• finally, in relation to the issues discussed, is there anything else you want to add or you think is important which should be included in this study?

## Competing interests

The author(s) declare that they have no competing interests.

## Authors' contributions

HL designed the study and carried out interviews and the data analysis. CS advised on study design, and collaborated on the data analysis.

Both wrote the paper.

## Pre-publication history

The pre-publication history for this paper can be accessed here:



## Figures and Tables

**Table 1:** Characteristics of study cohort (36 participants)

Training stage	13 students in Years 1 & 2; 16 students in Years 3 & 4; 7 students in Year 5
Gender	21 female, 15 male
Age	23 years (mean)
Ethnicity (self-described)	1 African-Asian, 1 Arab, 2 Bangladeshi, 2 Black, 1 Chinese, 1 Irish, 1 Jewish, 4 Indian, 2 Pakistani, 1 Persian, 20 'White'
Family status	33 single, 1 engaged, 2 married. None had children
Place of birth	30 UK, 1 other Europe, 5 outside Europe
Religion	9 Christian, 9 Muslim, 1 Hindu, 1 Jewish, 16 none
Entry to medical School	18 after school, 6 after a gap year, 3 one year off and other activities and 9 mature. (Seven obtained intercalated BSc during their time at medical school)
